# Evaluation of circulating transcript analysis (NETest) in small intestinal neuroendocrine neoplasms after surgical resection

**DOI:** 10.1007/s12020-020-02289-2

**Published:** 2020-04-14

**Authors:** Faidon-Marios Laskaratos, Man Liu, Anna Malczewska, Olagunju Ogunbiyi, Jennifer Watkins, Tu Vinh Luong, Dalvinder Mandair, Martyn Caplin, Christos Toumpanakis

**Affiliations:** 1 Centre for Gastroenterology, Neuroendocrine Tumour Unit, ENETS Centre of Excellence, Royal Free Hospital, London, UK; 2 Department of Gastroenterology, The First Affiliated Hospital, Sun Yat-Sen University, Guangzhou, China; 3 Department of Endocrinology and Neuroendocrine Tumors, Medical University of Silesia, Katowice, Poland; 4 Department of Surgery, Royal Free Hospital, London, UK; 5 Histopathology Department, Royal Free Hospital, London, UK

**Keywords:** Neuroendocrine tumour, Small bowel, Surgery, NETest

## Abstract

**Purpose:**

Surgical resection is the only effective curative strategy for small intestinal neuroendocrine neoplasms (SINENs). Nevertheless, the evaluation of residual disease and prediction of disease recurrence/progression remains a problematic issue.

**Methods:**

We evaluated 13 SINENs that underwent surgical resection of the primary tumour and/or mesenteric mass. Patients were divided in three groups: (a) Group 1: SINENs that underwent resection with curative intent, (b) Group 2: SINENs treated with resection in the setting of metastatic disease, which remained stable and (c) Group 3: SINENs treated with resection in the setting of metastatic disease, with evidence of any progression at follow-up. NETest and chromogranin A were measured pre-operatively and post-operatively during a 22-month median follow-up period and compared with imaging studies. NETest score <20% was determined as normal, 20–40% low, 41–79% intermediate and ≥80% high score.

**Results:**

NETest score was raised in all (100%) SINENs pre-operatively. Surgery with curative intent resulted in NETest score reduction from 78.25 ± 15.32 to 25.25 ± 1.75 (*p* < 0.05). Low NETest scores post-operatively were evident in all cases without clinical evidence of residual disease (Group 1). However, the low disease activity score suggested the presence of microscopic residual disease. In three cases (75%) with stable disease (Group 2) the NETest score was low consistent with indolent disease. In the progressive disease group (Group 3), a high NETest score was present in three cases (60%) and an intermediate NETest score in the remainder (40%).

**Conclusions:**

Blood NETest scores accurately identified SINENs and were significantly decreased by curative surgery. Monitoring NETest post-operatively may facilitate management by identifying the presence of residual/progressive disease.

## Introduction

Neuroendocrine neoplasms (NENs) are a heterogeneous group of malignancies that arise from neuroendocrine cells of the diffuse neuroendocrine system. The most common disease sites are the gastrointestinal tract, pancreas and the bronchopulmonary system [1]. Small intestinal neuroendocrine neoplasms (SINENs) arise in the jejunum and ileum and represent 26% of all diagnosed NENs [2–4]. SINENs often present late with extensive liver and nodal metastases due to the nonspecific nature of their symptomatology and lack of clinical recognition [2]. Nevertheless, resection of the primary tumour and mesenteric mass is usually advocated, since retrospective studies have noted that resection confers survival advantage by preventing local complications and controlling systemic symptoms. It should be noted, however, that these analyses are limited by selection bias [4]. Thereafter, regular follow-up using imaging and biomarkers is utilised to detect recurrence or disease progression at an earlier stage [3]. Imaging may be difficult to interpret post-operatively and current biomarkers such as chromogranin A (CgA) have limited efficacy [5]. In order to facilitate early identification of residual and/or recurrent disease, there therefore exists a critical unmet need to identify accurate and reliable biomarkers for NEN management [6]. The prediction of recurrence using histological and pathological criteria is unreliable since a pathologist has no information in respect of residual disease. Grading and staging are useful as general tools for stratification of disease but provide limited biological information to accurately predict tumour status [7]. In general, recurrence is unpredictable and early disease progression, especially of low-grade tumours (G1/G2) is difficult to predict [8]. A key unmet need in improving outcome is the early detection of recurrent and progressive disease and the timely initiation of treatment after surgical resection. Molecular markers in blood that identify disease presence and define progress would represent a significant advance in resolving this problem [5].

Current guidelines to evaluate tumour recurrence and disease progression recommend regular radiological examinations and biomarker evaluation during follow-up [1]. Functional imaging with somatostatin receptor-based strategies, such as ^68^Ga-somatostatin analogue (SSA) positron emission tomography (PET)/computed tomography (CT), has considerable value, but limited spatial resolution for the detection of small (<5 mm) lesions. CT and magnetic resonance imaging (MRI) also have difficulties in identifying marginal changes of tumour size [9].

Conventional secretory biomarkers such as CgA have previously been routinely used and were considered as appropriate biomarkers for NEN management [10]. However, over time it has become apparent that CgA assay(s) has numerous limitations and limited clinical utility [11]. These include its low sensitivity, specificity, poor laboratory metrics as well as nonspecific elevation caused by proton pump inhibitors and a variety of clinical conditions. A further limitation of CgA is that its measurement has little relevance to the biological processes that determine cell proliferation, invasion and metastasis [12]. As a consequence, most physicians rely predominantly on imaging studies and the overall clinical picture to guide patient management [13]. Based upon the subjective nature of clinical symptomatology, the limitations in imaging and the issues with CgA, there has been considerable enthusiasm to develop novel and more accurate NEN biomarkers.

Amongst a wide range of novel biomarkers, perhaps the most extensively studied is the NETest, a multigene circulating transcriptomic signature that has been shown to capture the multidimensionality of neuroendocrine neoplasia [14]. It has a sensitivity and specificity of >95 and >90% for diagnosis of disease and has been shown in a number of studies in gastroenteropancreatic and bronchopulmonary NENs (BPNENs) to be more accurate than CgA for monitoring disease progress [15–17]. Independent analysis of the NETest liquid biopsy strategy has shown promising results, in terms of its ability to define the completeness of surgical resection, identify early recurrence or progressive disease after surgery and determine efficacy of treatment in pancreatic and lung NETs [14].

This prospective surgical cohort study aimed to evaluate the NETest as a biomarker for the assessment of surgical resection, the detection of residual disease and tumour progression after primary resection in SINENs. The study provides real-life data with extended clinical follow-up of these patients using imaging studies and conventional secretory biomarkers.

## Material and methods

### Patients

Thirteen patients, who were operated on for SINENs at the Royal Free Hospital between 2017 and 2018, were studied. All patients provided informed consent. Imaging studies to evaluate disease status included CT, MRI and ^68^Ga-SSA-PET/CT. The majority of patients had a ^68^Ga PET/CT at baseline (before surgery) for staging of the disease, as well as 3 months after surgery to evaluate the presence of residual/recurrent disease. Standard cross-sectional imaging with CT or MRI was performed every 4–6 months during the follow-up period to monitor disease status and exclude recurrence and ^68^Ga PET/CT was usually performed every year. The precise details of the imaging modalities used for the study cohort are provided in Online Resource 1. Disease was considered stable, if no radiological progression was noted and progressive, if there was evidence of any radiological progression, using RECIST 1.1 criteria. The study group demographics and clinicopathological characteristics are shown in Tables 1 and 2.

**Table 1:** Patient and tumour characteristics

		Sex	Age	Tumour grade	Differentiation	Tumour stage	Metastatic lesions from midgut NETs
Group 1 (*n* = 4)	1	M	63	G1	Well differentiated	pT3N2M1	Mesenteric nodes, pancreatic metastasis
	2	M	48	G2	Well differentiated	pT2NxMx	None
	3	M	66	G2	Well differentiated	pT3N1MX	Mesenteric nodes
	4	M	67	G1	Well differentiated	pT2N1MX	None
Group 2 (*n* = 4)	1	M	58	G1	Well differentiated	pT4N1M1	Mesenteric mass
	2	M	65	G2	Well differentiated	pT2N1Mx	Mesenteric nodes, liver
	3	F	79	G1	Well differentiated	pT2N1M1	Mesenteric mass, liver
	4	F	66	G2	Well differentiated	pT4N1MX	Mesenteric mass, liver
Group 3 (*n* = 5)	1	M	55	G1	Well differentiated	pT4N1M1	Mesenteric mass and adjacent lymph nodes
	2	M	63	G1	Well differentiated	pT4N1M1	Liver
	3	M	59	G1	Well differentiated	pT4N1Mx	Liver, bone
	4	M	73	G1	Well differentiated	pT4N1M1	Mesenteric mass, liver
	5	F	69	G2	Well differentiated	pT4N1M1	Mesenteric nodes

**Table 2:** Surgical resection characteristics

Total number (*n* = 13)		Surgery	Resection	Tumour size	Lymph node involvement	Vascular invasion
Group 1 (*n* = 4)	1	Right hemicolectomy; Distal pancreatectomy	R0	Primary tumour: 1.5 cm; Mesenteric mass: 2 cm	2/23	V1
	2	Small bowel resection	R0	Tumour: 1.7 cm	0/1	V0
	3	Right hemicolectomy	R1	Primary tumour: 2.3 cm; Mesenteric mass: 3.7 cm	0/20	V1
	4	Right hemicolectomy	R0	Primary tumour: 2.9 cm	7/21	V1
Group 2 (*n* = 4)	1	Right hemicolectomy	R1	Primary tumour: 2.5 cm; Mesenteric mass: 2.5 cm	9/41	V1
	2	Right hemicolectomy	R0	Primary tumour: 1.3 cm	3/36	V0
	3	Right hemicolectomy; Omentectomy	R0	Primary tumour: 2.2 cm; Mesenteric mass: 6.6 cm	3/11	V0
	4	Right hemicolectomy	R0	Primary tumour: 2 cm	3/14	V1
Group 3 (*n* = 5)	1	Right hemicolectomy; Small bowel resection	R1	Primary tumour: 3.2 cm	9/20	V1
	2	Right hemicolectomy	R0	Primary tumour: 1.8 cm	4/15	V1
	3	Right hemicolectomy	R1	Primary tumour: 7 cm	4/7	V1
	4	Right hemicolectomy	R0	Primary tumour: 5 cm	2/6	V1
	5	Right hemicolectomy	R1	Primary tumour: 1.5 cm; Mesenteric mass: 3 cm	9/35	V1

### Sample collection

Whole blood for NETest measurement was collected at baseline (the day before surgery) and thereafter at clinically defined points during the follow-up. Blood samples (10 ml) were collected in ethylenediaminetetraacetic acid (EDTA) tubes (BD Vacutainer Venous Blood Collection Tubes, BD Diagnostics, Franklin, NJ). Aliquots of whole blood were stored at −80 °C within 2 h of collection (samples immediately stored on ice/4 °C after sampling) for PCR-based studies.

### NETest measurement

A two-step protocol [mRNA isolation, complementary DNA production and polymerase chain reaction (PCR)] was used [18]. Transcripts (mRNA) were isolated from EDTA-collected whole blood samples. PCR values of the 51 markers were normalized to housekeeping genes, and expression was quantified against a population control. Expression levels were converted to an activity score ranging from 0 (low activity) to 100 (high activity) [19].

### Chromogranin A enzyme-linked immunosorbent assay

CgA was measured using DAKO ELISA kit (K0025, DAKO North America, Inc, Carpinteria, CA). A cutoff of 27 U/L defined the upper limit of normal (ULN).

### Statistical analysis

Statistical analysis was performed using Prism 6.0 for Windows (GraphPad Software, La Jolla, CA; www.graphpad.com) and SPSS Statistics for Windows version 23.0 (IBM Corp., Armink, NY). Data were expressed as mean values ± standard error of the mean. Statistical analysis was performed using unpaired Student’s *t* tests or Welch’s *t* tests to assess the differences between the study groups. For correlation analysis, *p* values and correlation coefficients (*r*) were calculated using Pearson’s correlation test. Results were considered significant at *p* < 0.05. To investigate the prognostic value of NETest and CgA in predicting disease progression, SPSS was used to perform receiver operating characteristics curve analyses and the sensitivity, specificity and area under the curve were calculated.

## Results

### Patient demographics and follow-up

Patients’ and tumour characteristics are presented in Table 1, and surgical resection details are presented in Table 2. In total, 13 patients with SINENs who underwent surgical resection of primary tumour (with or without mesenteric mass) were included. The patient cohort consisted of nine males and four females, with a median age of 65 years (range, 48–79 years). All patients were diagnosed with well-differentiated SINENs, and eight patients had G1 NENs, while five patients had G2 NENs. Patients were divided in three groups. Group 1 (*n* = 4): resection with curative intent and no clinical evidence of recurrence at follow-up; Group 2 (*n* = 4): resection in the setting of metastatic disease, which remained stable at follow-up; Group 3 (*n* = 5): resection in the setting of metastatic disease, with evidence of progression at follow-up. The median follow-up period in this study was 22 months (range: 13–28 months). Some of the patients in Groups 2 and 3 were on somatostatin analogue therapy before surgical resection, while none of the patients in Group 1 were receiving medical therapy at baseline. Details of previous therapies for the study cohort are provided in Online Resource 2.

### Baseline level of NETest score and CgA before surgery

**Fig. 1:**
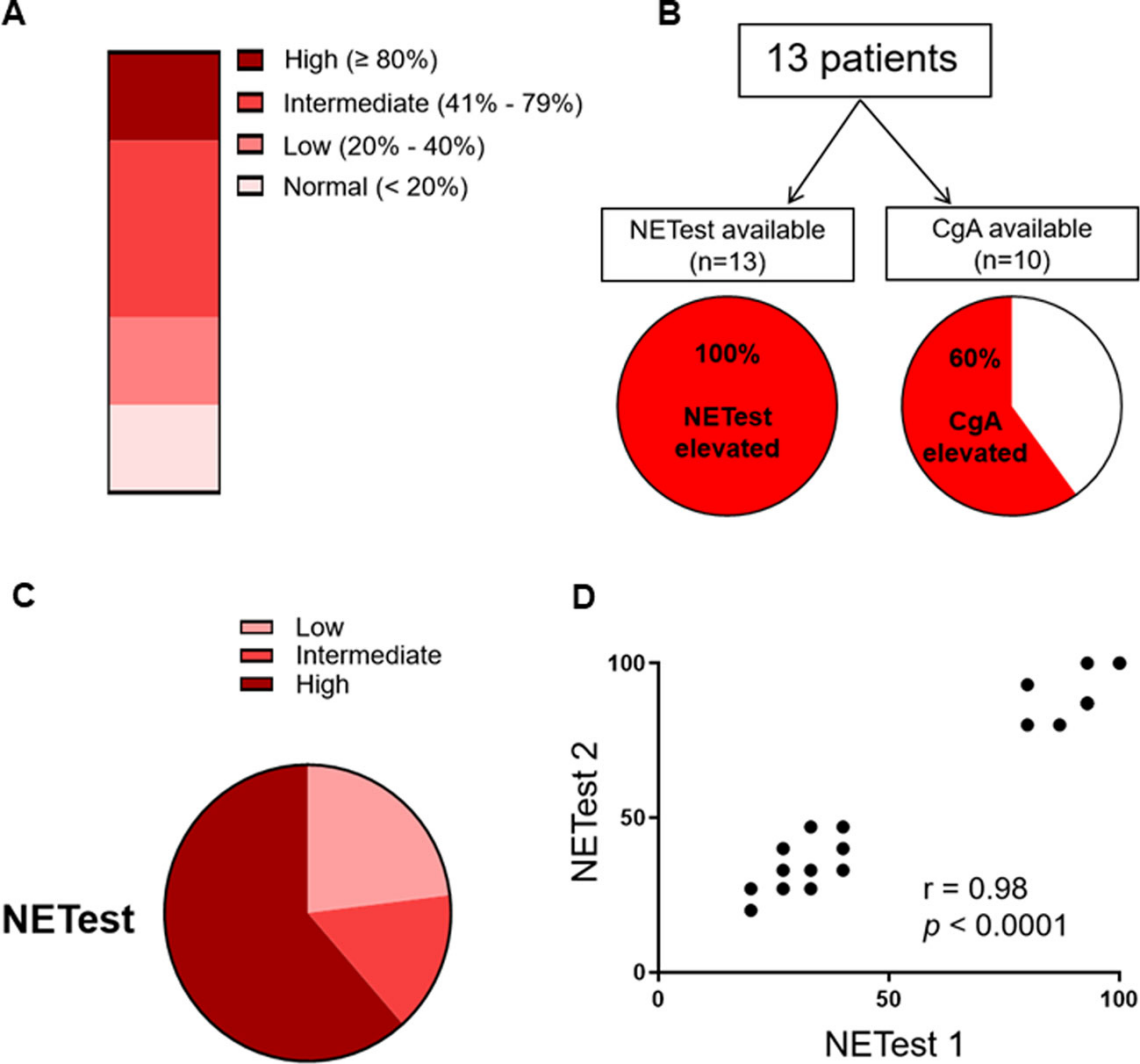
Basal level of NETest score and CgA in the pre-surgery stage. **a** Scale range of NETest-indicated disease activity. **b** The NETest was positive in all 13 (100%) and CgA was positive in 6 of the 10 patients (60%). **c** The distribution of pre-surgery NETest score in this study cohort. **d** Reproducibility of the NETest. There was a strong and highly significant correlation between NETest values in two separate blood samples collected at the same time point in patients with midgut NETs (*r* = 0.98, *p* < 0.0001)

Baseline levels of NETest scores were evaluated in patients with SINENs before surgery. Gene expression data are used in the calculation of the NETest score, ranging from 0 to 100 that reflects disease activity. The ULN is 20 [20], scores of 20–40 are associated with low disease activity, 41–79 represent intermediate disease activity and ≥80 reflect high disease activity (Fig. 1a) [17]. NETest scores were elevated in all patients (100%) before surgery (71.9 ± 7.8) (Fig. 1b). Most patients (*n* = 8, 61.5%) had high NETest scores, three patients (23.1%) had low NETest scores and two patients (15.4%) had intermediate NETest scores (Fig. 1c). Reproducibility of the NETest was evaluated by dual sample comparison. There was a strong and highly significant correlation between NETest values in two separate blood samples collected at the same time point in patients with SINENs (*r* = 0.98, *p* < 0.0001) (Fig. 1d).

We also evaluated CgA levels in patients with SINENs before surgery. CgA levels were available in 10 of the 13 patients (81.7 ± 28.39 U/L; ULN: 27 U/L). Only 6 of the 10 subjects displayed elevated CgA levels, whereas CgA levels of the remaining 4 cases were within normal range (Fig. 1b). This suggests that the presence of NET disease was more effectively identified by the NETest (13/13, 100%) compared with CgA (6/10, 60%) (*p* = 0.024).

### Changes in NETest and CgA levels after surgical resection in patients with SINENs

#### NETest levels

**Fig. 2:**
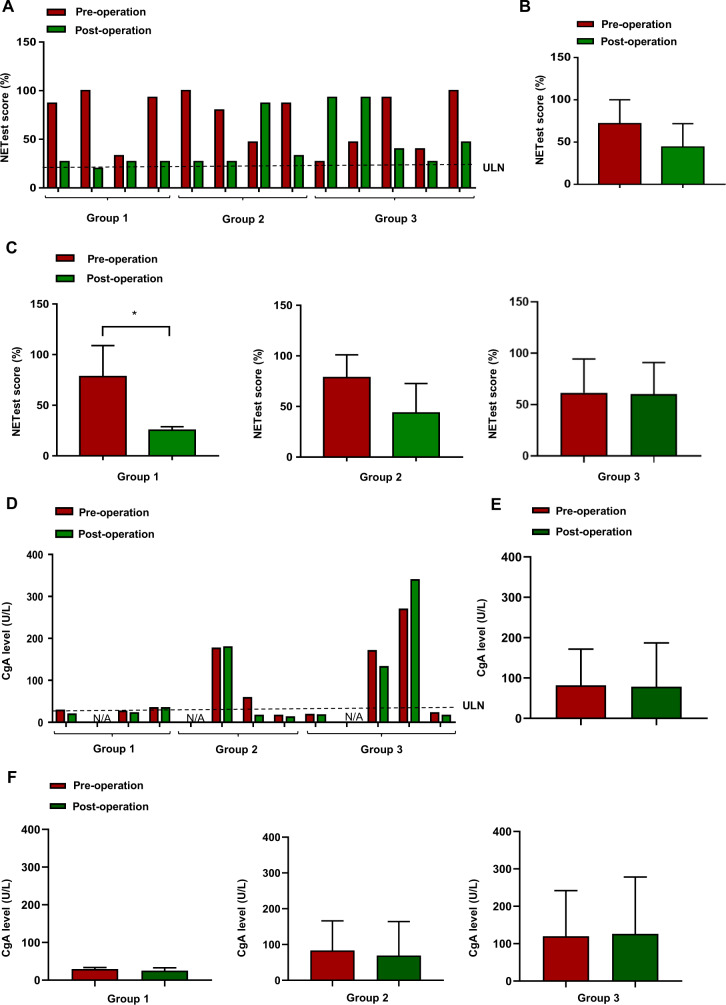
Effect of tumour resection surgery on NETest and CgA levels in patients with midgut NETs. **a** Individual NETest scores (pre- and postoperative levels) in groups 1, 2 and 3. **b** Pre- and postoperative NETest scores in the entire study cohort (*n* = 13, *p* = 0.067). **c** Pre- and postoperative NETest scores in groups 1, 2 and 3 (Group 1: *n* = 4, *p* = 0.047; Group 2: *n* = 4, *p* = 0.2623; Group 3: *n* = 5, *p* = 0.958). *p ≤ 0.05. **d** Pre- and postoperative CgA measurements in individual patients of groups 1, 2 and 3. **e** Pre- and post-operative CgA measurements in the entire patient cohort. **f** Pre- and postoperative CgA measurements in each of the groups 1, 2 and 3

Individual NETest scores (pre- and post-surgery levels) were assessed and in 10 out of 13 (76.9%) patients, NETest scores decreased after surgery consistent with a reduction in tumour burden (Fig. 2a). The mean post-surgery NETest score was 44.2 ± 7.6, which was lower than the mean score (71.9 ± 7.8, *p* = 0.067) in the pre-surgery stage (Fig. 2b). We further separately assessed alterations of NETest score in each group. Decreases in NETest after surgery were statistically significant in Group 1 (pre-surgery: 78.25 ± 15.32; post-surgery: 25.25 ± 1.75; *p* = 0.047), but not significant in Group 2 (pre-surgery: 78.50 ± 11.29; post-surgery: 43.50 ± 14.57; *p* = 0.262) and Group 3 (pre-surgery: 61.40 ± 14.73; post-surgery: 60.00 ± 13.85; *p* = 0.958) (Fig. 2c). Therefore, the NETest score was significantly decreased after curative surgery, since patients in Group 1 mainly had small volume of disease that was removed during surgery, in comparison to the setting of metastatic disease (Groups 2 and 3), where the primary tumour and mesenteric mass resected accounted for a small proportion of the overall disease volume (usually liver-predominant).

#### CgA Levels

Individual CgA levels (pre- and post-surgery levels) were also assessed and 7 out of 10 patients (70%) exhibited a slight reduction in CgA levels after surgery (Fig. 2d). The mean post-surgery CgA level was 78.6 ± 34.25 U/L, which was slightly (but not significantly) decreased compared with the mean level (81.7 ± 28.39 U/L, *p* = 0.752) before surgery (Fig. 2e). Decreases in CgA levels after surgery were not significant in any groups: Group 1 (pre-surgery: 29.33 ± 2.404 U/L; post-surgery: 25 ± 4.583 U/L; *p* = 0.238), Group 2 (pre-surgery: 83.33 ± 47.89 U/L; post-surgery: 69.00 ± 55.01 U/L; *p* = 0.413) and Group 3 (pre-surgery: 119.80 ± 61.04 U/L; post-surgery: 126.00 ± 76.04 U/L; *p* = 0.802) (Fig. 2f). Therefore, CgA levels were not significantly altered by surgical treatment (i.e. after reduction in disease volume).

### Follow-up assessments of NETest and CgA in post-surgery stage

#### NETest levels

**Fig. 3:**
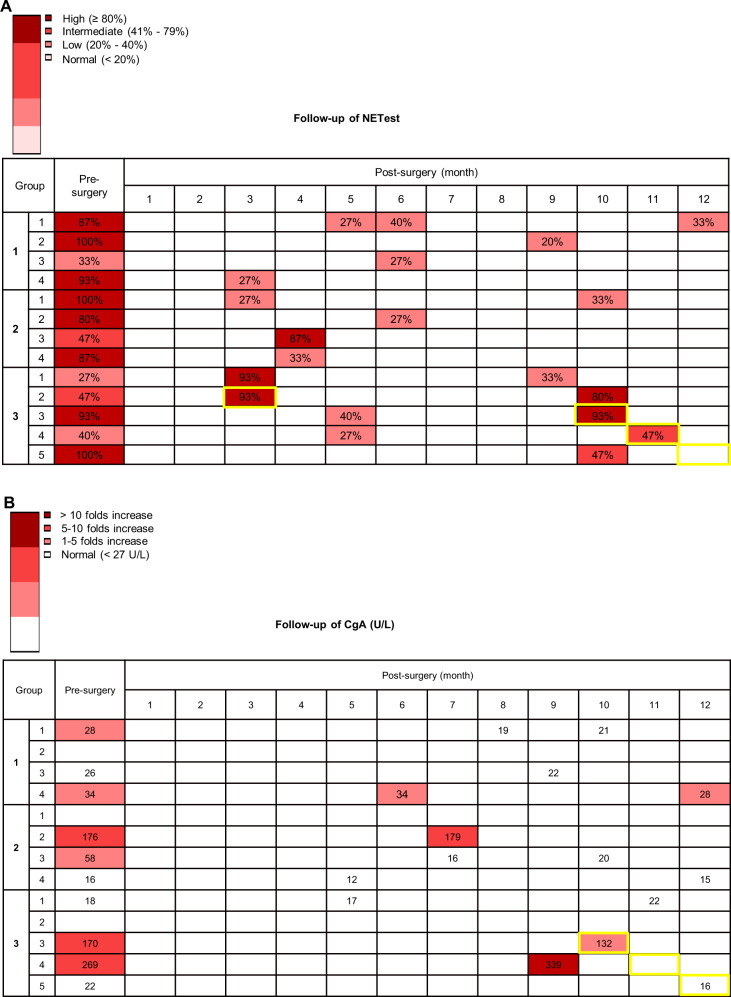
Follow-up assessments of NETest and CgA. **a** The recorded NETest scores at indicated time points of each patient are displayed. **b** The recorded CgA levels at indicated time points of each patient are displayed. Yellow framed time points indicate time of disease progression

The recorded NETest scores at indicated time points for each patient are included in Fig. 3a. Of the four patients in Group 1, no patients developed disease recurrence by the time of the last follow-up, in keeping with their low NETest scores in the post-surgery stage. However, the presence of low-level disease activity in the NETest scores of these patients is concerning and is consistent with molecular evidence of image-negative disease after surgery [21]. In Group 2, three patients had low NETest scores in the post-surgery stage, in keeping with their stable disease status. One exception is that, in patient #3 (Group 2), the NETest score increased after resection (from 47 to 87%), but the disease was considered stable during the last follow-up (19 month after the resection). We anticipate that a more extended follow-up period would be required before we could exclude a delayed progressive event. In Group 3, progressive disease was detected in five patients by imaging (Gallium-68 PET/CT; CT; MRI) during the post-surgery follow-up. In the post-surgery stage, three patients in Group 3 had high NETest scores, while two patients had intermediate scores. The NETest scores accurately correlated with progression.

#### CgA levels

The recorded CgA levels at indicated time points of each patient are shown in Fig. 3b. In Group 1, patient #1 and #3 had normal CgA levels in the post-surgery stage, while patient #4 had slightly increased CgA levels in the post-surgery stage. In Group 2, patient #2 had more than fivefold increase of CgA in the post-surgery stage, but patients #3 and #4 had normal CgA levels in the post-surgery stage, despite the presence of metastatic disease. In Group 3, patient #3 and #4 had high levels of post-surgery CgA levels, but patient #1 and #5 had normal CgA levels in the post-surgery stage, which was not consistent with their disease progression status. Overall, post-surgery CgA levels were clinically non-informative.

### Individual evaluation of NETest and CgA in patients with disease progression

Patient #1 (Group 3) had a NETest score of 93% detected at 3 months after his surgery. Although the NETest score dropped to 33% at 9 months after the surgery, the patient still developed progressive disease at 24 months. These results suggest that high NETest scores soon after surgery may be a risk factor for disease progression during long-term follow-up and the progressive event may sometimes occur more than 1 year later. In contrast, CgA levels remained in the normal range. This was 17 U/L at 5 months and 22 U/L at 10 months after the surgery, and there was no correlation with disease status.

Patient #2 (Group 3) had a NETest score of 93% detected at 3 months after his surgery. Meanwhile, the patient developed progressive disease at 3 months after the surgery. The NETest score remained at a high level (80%) at 10 months after the surgery. The disease progression was consistent with the high NETest measurements.

Patient #3 (Group 3) had a NETest score of 40% detected at 5 months after surgery. The patient developed disease progression at 10 months after his operation, and the NETest score increased from 40 to 93% at this time point. The increased NETest score was in accordance with the progressed disease status. CgA level was also at a high level of 132 U/L at the time of disease progression.

Patient #4 (Group 3) had NETest score of 27% detected at 5 months after his surgery. The patient developed disease progression at 11 months after surgery, and the NETest score increased from 27 to 47% at that time point. CgA level was also elevated at 339 U/L before the time of disease progression.

Patient #5 (Group 3) had a NETest score of 47% detected at 10 months after her surgery. The patient developed disease progression at 12 months after her surgery, but CgA level was in the normal range at the time of disease progression.

### Evaluation of NETest and CgA in identifying disease status

**Fig. 4:**
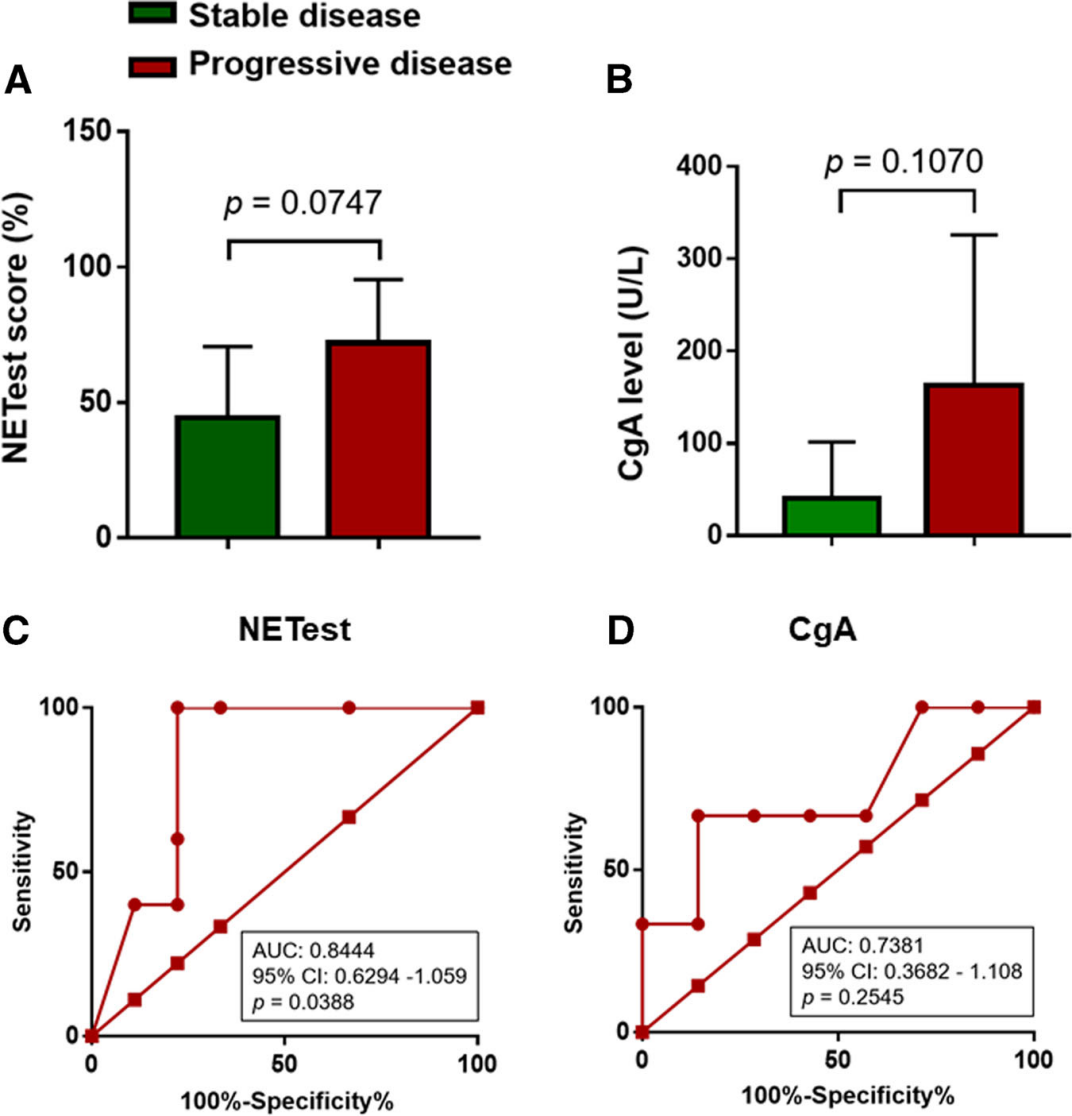
Evaluation of NETest and CgA in identifying disease status. Comparison of NETest scores (**a**) or CgA levels (**b**) detected in stable disease or progressive disease period. ROC analysis and AUC were used to assess the prognostic capacity of NETest score (**c**) or CgA (**d**) to predict disease progression

The NETest scores or CgA levels detected within 2 months of the time of disease progression were compared with those detected during the stable disease period. The NETest was elevated in progressive disease (72 ± 10.48) compared with stable disease (44.44 ± 8.74, *p* = 0.075; Fig. 4a). CgA was also elevated in progressive disease (162.3 ± 94.47 U/L) compared with stable disease (40.14 ± 23.18, *p* = 0.107; Fig. 4b). The AUROC of NETest for differentiating progressive disease from stable disease was 0.844 (95% CI 0.6294–1.059) (*p* = 0.039; Fig. 4c). The optimum cutoff value of NETest score to predict progressive disease was >43.5%. The sensitivity and specificity were 100% and 77.78%, respectively. The AUROC of CgA for differentiating progressive disease from stable disease was 0.738 (95% CI 0.3682–1.108) (*p* = 0.255; Fig. 4d).

## Discussion

One of the major challenges in the management of NENs is that neither anatomical imaging modalities nor conventional blood biomarkers such as CgA can accurately prognosticate tumour behaviour [22]. Furthermore, repeated invasive biopsy increases risk is uncomfortable and is, in many circumstances, not practical [17]. Therefore, the development of accurate non-invasive biomarkers that define disease status and behaviour is a critical unmet need. In other oncological disciplines, considerable attention has been focused on the development of liquid biopsy as a tool to identify disease status in real time [23–26]. Early diagnosis of SINENs and identification of recurrent or residual disease progression after surgical resection is key to timely manage patients with SINENs.

A key issue in NET biomarkers has been the fact that all are monoanalytes and represent tumour secretory function. The limitations of this are twofold. First, that secretory activity of tumours is not a dominant determination of disease malignancy, progression and metastasis. Second, a monoanalyte measurement is, by definition, unidimensional and does not capture the diverse biological processes of a tumour cell that a multigene assay measures. Thus, in comparison to monoanalyte measurements, the transcriptome-based NETest is a multianalyte (51 genes) biomarker that has shown promising results and is more reliable in diagnosis and defining NEN disease status [27–32].

In this study, the clinical utility of the NETest as a biomarker in SINENs treated with surgical resection of the primary tumour and/or mesenteric mass was evaluated. NETest scores were elevated in all SINEN patients (100%), but CgA was only elevated in 60% of the patients pre-operatively. This is in accordance with one previous study [33], where the accuracy of NETest for detection of small bowel NENs was reported to be 93%, but CgA was only positive in 54% NENs. As a diagnostic marker, the NETest was significantly more sensitive than CgA for midgut NENs [16, 33].

We further determined changes in NETest and CgA levels after surgical resection of the primary tumour. NETest scores were significantly decreased after curative surgery (Group 1), but CgA decrease was insignificant in this subgroup. Peritoneal metastases and residual mesenteric disease were not evident on functional and anatomical studies during the follow-up period. In addition, in the operation notes, there was no record of unresected pathological mesenteric lymph nodes or peritoneal metastases. However, the presence of low-level disease activity (a low but positive NETest score) after surgery is concerning and suggests the presence of microscopic residual disease that may become apparent during extended follow-up [21]. It is of interest to note that previous studies have reported that blood measurement of NETest can effectively define the completeness of operative resection in BPNENs and pancreatic NENs [16, 34–36].

More importantly, the NETest, but not CgA, could significantly differentiate progressive disease from stable disease in the post-surgery stage. In our study, NETest scores >43.5% significantly correlated with progressive disease. Such an effective prediction of disease progression would facilitate stratification of the patients who are at higher risk. We anticipate that this molecular genomic strategy will identify individuals (elevated scores) who would benefit from closer monitoring, in comparison to others (low scores) who could be monitored less frequently. In previous studies, elevated NETest scores have been noted to be associated with poor progression-free survival [14, 32, 35]. It is interesting, however, that in some cases (such as patient #1) the high NETest score predicted disease progression at a very early stage (>12 months prior to the progressive event), while in other cases (such as patients #3 and #4) the progression coincided with a significant (approximately twofold) elevation of the NETest to intermediate/high activity range. We believe that significant elevations in the NETest score reflect changes in tumour behaviour at a cellular level. Although sometimes these changes will become evident at radiology at the time of the NETest elevation, in more indolent tumours it may take some time for changes in the biology of the tumour to manifest as radiologically confirmed progressive disease and this may become evident many months later. This is in keeping with previous publications which have shown the predictive ability of the NETest to identify disease progression at a very early stage [32].

We determined a cutoff of 43.5% for the NETest in predicting disease progression in this surgical series. The sensitivity and specificity were 100% and 77.78%, respectively. In agreement with previously published literature, NETest score >40 has been demonstrated to differentiate those with low risk of disease from those with a moderate or high risk of disease activity, and has been identified to be prognostic for disease progression [14, 30, 32]. In contrast, NETest scores <40 in those with stable disease were consistent with disease stability [14, 32].

In conclusion, our study provides real-life data of NETest utilisation in a prospective cohort of SINENs treated with surgical resection. However, this study does have some limitations. The sample size for each group is small and the follow‐up duration of patients may not be long enough. As recurrence typically develops within the first 5 years after surgery [35], a median follow-up of 19 months in group 1 may not be adequate to identify the patients who will eventually develop recurrence. Certainly, extended follow-up is warranted in view of the low-level disease activity detected by the NETest after surgery in these cases. It is possible that information generated by “gene cluster” analysis of a NETest score will provide additional information to better define the risk of an individual tumour [19]. We anticipate that with the growing body of evidence demonstrating the clinical utility of liquid biopsy that, in the near future, the NETest may prove a useful adjunct to the clinical management of post-surgical patients with SINENs. Larger prospective studies are warranted to comprehensively explore the utility of the NETest in the identification of post-operative residual NENs disease progression or recurrence, and to facilitate a better personalization of post-operative care for SINENs. Our study demonstrates that a multianalyte blood test is a more effective NET biomarker than the measurement of the monoanalyte CgA.

## Supplementary material

Online Resource 1

Online Resource 2

## Data Availability

All data generated or analysed during this study are included in this published article [and its supplementary information files].
