# Supplementary material for: Evaluation of circulating transcript analysis (NETest) in small intestinal neuroendocrine neoplasms after surgical resection
Source: Endocrine. 2020 Apr 14;69(2):430–40. doi: 10.1007/s12020-020-02289-2 (PMC7392928; doi:10.1007/s12020-020-02289-2)
Supplement: Supplementary file 1 [file 12020_2020_2289_MOESM1_ESM.doc]

Online Resource 1. Imaging modalities used for staging of disease at baseline (before surgery) and evaluation of disease status at various time points during follow-up

		**Surgery date**	Baseline imaging	**Follow-up imaging**											
Group 1	1	Surgery	Ga68 PET/CT	Ga68 PET/CT	CT	Ga68 PET/CT	CT	CT							
	Date	13/04/2017	14/12/2016	07/11/2017	23/04/2018	21/11/2018	29/04/2019	01/11/2019							
	2	Surgery	No	Ga68 PET/CT	CT Thorax and MRI abdomen, liver and spleen	CT Thorax, MRI Pelvis, abdomen with contrast	CT abdomen, liver with contrast	CT Thorax; MRI Pelvis, liver with contrast,							
	Date	24/04/2017	N/A	02/08/2017	22/01/2018	21/08/2018	07/05/2019	18/10/2019							
	3	Surgery	Ga68 PET/CT	Ga68 PET/CT											
	Date	10/01/2018	20/10/2017	22/06/2018											
	4	Surgery	Ga68 PET/CT	CT Abdomen and pelvis with contrast	Ga68 PET/CT	Ga68 PET/CT	CT	CT	18FDG PET/CT						
	Date	22/11/2017	14/09/2017	19/02/2018	09/03/2018	10/08/2018	14/08/2018	16/09/2019	12/10/2019						
Group 2	1	Surgery	Ga68 PET/CT	Ga68 PET/CT; MRI liver	Ga68 PET/CT	Ga68 PET/CT	Ga68 PET/CT								
	Date	04/05/2017	06/04/2017	09/08/2017	30/08/2018	15/03/2019	19/09/2019								
	2	Surgery	CT Thorax abdomen pelvis with contrast	Ga68 PET/CT; CT Thorax abdomen pelvis with contrast	CT Thorax abdomen pelvis with contrast	CT Thorax abdomen pelvis with contrast	MRI liver with contrast	Ga68 PET/CT	MRI abdomen, pelvis, liver with constrast	CT Thorax, abdomen, liver, pelvis with contrast	Ga68 PET/CT				
	Date	01/10/2017	12/09/2017	31/01/2018	27/04/2018	10/08/2018	12/09/2018	13/09/2018	06/03/2019	17/09/2019	18/11/2019				
	3	Surgery	Octreotide scan	MRI pelvis, liver with constrast	Ga68 PET/CT	MRI pelvis, liver with contrast	MRI head with contrast	MRI head with contrast	MRI abdomen, pelvis with contrast	Ga68 PET/CT	CT Thorax with contrast	MRI pelvis, liver with contrast	MRI head with contrast	MRI pelvis, liver with contrast	
	Date	01/02/2018	06/12/2017	26/03/2018	12/06/2018	16/07/2018	02/08/2018	09/11/2018	06/03/2019	15/03/2019	26/03/2019	30/09/2019	19/12/2019	20/02/2020	
	4	Surgery	Ga68 PET/CT	Ga68 PET/CT	CT Thorax, abdomen, liver, pelvis with contrast	MRI liver and spleen with contrast	CT thorax with contrast	MRI pelvis, abdomen, liver and spleen with contrast	MRI pelvis, liver and spleen with contrast	CT thorax with contrast; MRI liver with contrast	CT thorax with contrast	MRI pelvis, liver and spleen with contrast	Ga68 PET/CT	MRI spine lumbar and sacral	CT thorax; MRI abdomen, pelvis with contrast
	Date	29/06/2017	05/05/2017	29/09/2017	30/09/2017	07/11/2017	01/05/2018	28/06/2018	27/11/2018	27/11/2018	29/03/2019	01/04/2019	11/04/2019	10/05/2019	27/06/2019
Group 3	1	Surgery	Ga68 PET/CT	Ga68 PET/CT	Ga68 PET/CT	Ga68 PET/CT	Ga68 PET/CT	MRI pelvis, liver with contrast; CT thorax							
	Date	01/05/2017	17/03/2017	25/08/2017	16/02/2018	01/09/2018	04/04/2019	15/10/2019							
	2	Surgery	Ga68 PET/CT	Ga68 PET/CT; CT thorax, abdomen and pelvis with contrast; CT liver dual phase	18F-FDG PET/CT	MRI liver with contrast; CT thorax with contrast	CT thorax with contrast	MRI abdomen, pelvis with contrast	CT thorax	MRI abdomen, pelvis with contrast	MRI pelvis, liver with contrast				
	3	Surgery	Ga68 PET/CT	CT thorax, abdomen, pelvis with contrast	CT thorax, abdomen, pelvis with contrast; CT liver dual phase	CT thorax, abdomen, pelvis with contrast; CT liver dual phase	Ga68 PET/CT	18F-FDG PET/CT	MRI spine thoracolumbar	MRI spine lumbar, sacral	CT thorax, abdomen, pelvis with contrast; Liver dual phase	CT thorax, abdomen, pelvis with contrast; CT liver dual phase	CT thorax, abdomen, pelvis with contrast; CT liver dual phase		
	Date	17/06/2017	06/01/2017	14/10/2017	24/03/2018	29/07/2018	14/08/2018	10/09/2018	27/10/2018	22/11/2018	31/01/2019	30/05/2019	23/08/2019		
	4	Surgery	Ga68 PET/CT	CT thorax, abdomen, pelvis with contrast	Ga68 PET/CT	CT thorax, abdomen, pelvis with contrast	Ga68 PET/CT	CT Thorax, abdomen, pelvis with constrast; Liver dual phase	CT Thorax, abdomen, pelvis with contrast						
	Date	01/07/2017	02/06/2017	04/10/2017	06/10/2017	29/05/2018	23/08/2018	10/10/2018	18/04/2019						
							CT thorax, [illegible]								
