# Supplementary material for: Evaluation of circulating transcript analysis (NETest) in small intestinal neuroendocrine neoplasms after surgical resection
Source: Endocrine. 2020 Apr 14;69(2):430–40. doi: 10.1007/s12020-020-02289-2 (PMC7392928; doi:10.1007/s12020-020-02289-2)
Supplement: Supplementary file 2 [file 12020_2020_2289_MOESM2_ESM.doc]

Online Resource 2. Somatostatin analogue (SSA) therapy at baseline (before surgery) in patients included in the study. The dosage, injection interval and date of treatment initiation are provided for patients in each group.
